# Leukemia inhibitory factor protects the lung during respiratory syncytial viral infection

**DOI:** 10.1186/s12865-014-0041-4

**Published:** 2014-10-03

**Authors:** Robert F Foronjy, Abdoulaye J Dabo, Neville Cummins, Patrick Geraghty

**Affiliations:** Mount Sinai Roosevelt Hospital, Mt. Sinai Health System, Division of Pulmonary and Critical Care Medicine, New York, NY USA

**Keywords:** Respiratory syncytial virus, Immune response, Pathogen recognition receptors, Gene expression

## Abstract

**Background:**

Respiratory syncytial virus (RSV) infects the lung epithelium where it stimulates the production of numerous host cytokines that are associated with disease burden and acute lung injury. Characterizing the host cytokine response to RSV infection, the regulation of host cytokines and the impact of neutralizing an RSV-inducible cytokine during infection were undertaken in this study.

**Methods:**

A549, primary human small airway epithelial (SAE) cells and wild-type, TIR-domain-containing adapter-inducing interferon-β (*Trif*) and mitochondrial antiviral-signaling protein *(Mavs*) knockout (KO) mice were infected with RSV and cytokine responses were investigated by ELISA, multiplex analysis and qPCR. Neutralizing anti-leukemia inhibitory factor (LIF) IgG or control IgG was administered to a group of wild-type animals prior to RSV infection.

**Results and discussion:**

RSV-infected A549 and SAE cells release a network of cytokines, including newly identified RSV-inducible cytokines LIF, migration inhibitory factor (MIF), stem cell factor (SCF), CCL27, CXCL12 and stem cell growth factor beta (SCGF-β). These RSV-inducible cytokines were also observed in the airways of mice during an infection. To identify the regulation of RSV inducible cytokines, *Mavs* and *Trif* deficient animals were infected with RSV. *In vivo* induction of airway IL-1β, IL-4, IL-5, IL-6, IL-12(p40), IFN-γ, CCL2, CCL5, CCL3, CXCL1, IP-10/CXCL10, IL-22, MIG/CXCL9 and MIF were dependent on *Mavs* expression in mice. Loss of *Trif* expression in mice altered the RSV induction of IL-1β, IL-5, CXCL12, MIF, LIF, CXCL12 and IFN-γ. Silencing of retinoic acid–inducible gene-1 (RIG-I) expression in A549 cells had a greater impact on RSV-inducible cytokines than melanoma differentiation-associated protein 5 (MDA5) and laboratory of genetics and physiology 2 (LGP2), and Trif expression. To evaluate the role of LIF in the airways during RSV infection, animals were treated with neutralizing anti-LIF IgG, which enhanced RSV pathology observed with increased airspace protein content, apoptosis and airway hyperresponsiveness compared to control IgG treatment.

**Conclusions:**

RSV infection in the epithelium induces a network of immune factors to counter infection, primarily in a RIG-I dependent manner. Expression of LIF protects the lung from lung injury and enhanced pathology during RSV infection.

**Electronic supplementary material:**

The online version of this article (doi:10.1186/s12865-014-0041-4) contains supplementary material, which is available to authorized users.

## Background

Respiratory syncytial virus (RSV) is a major respiratory pathogen, which typically infects the airway epithelium [1]. Those infected with RSV, usually infants, the elderly and immunocompromised patients but also healthy adults [2,3], develop mild to severe cough and dyspnea [4]. Wheezing and asthma symptoms are observed following severe RSV lower respiratory tract infection [5]. RSV infection occur frequently in the respiratory tract of individuals with an underlying lung disease, such chronic obstructive pulmonary disease (COPD) [6]. A range of inflammatory responses are associated with RSV infection, such as the induction of proinflammatory cytokines, chemokines and growth factors, such as GROα/CXCL1, IL-1β, IL1-RA, IL-2, IL-6, IL-7, CXCL8/IL-8, IL-9, CXCL10/IP-10, CXCL11, IL-15, IL-18, CCL2/MCP-1, CCL3/MIP-1α, CCL4/MIP-1β, IFN-γ, CCL5/RANTES, epidermal growth factor (EGF), hepatocyte growth factor (HGF), granulocyte colony-stimulating factor (G-CSF), GM-CSF, fibroblast growth factor (FGF), vascular endothelial cell growth factor (VEGF) and tumor necrosis factor-α (TNF-α) [1,7-16]. The importance of this cytokine response is underscored by the fact that increased levels of IL-1β, IL1-RA, IL-6, IL-7, IL-8, G-CSF, EGF and HGF are associated with RSV infection severity [10,15]. Others have found that SNPs in IL-19, IL-20, MUC5AC, TNFRSF1B, C3, CTLA4, CXCL9, IL4R, and IL-7 genes are associated with recurrent wheezing following RSV infections [17]. Although RSV-induced cytokines have been studied extensively, the signaling mechanisms that regulate their expression and whether specific host cytokines contribute to airway injury or inflammation resolution remain incompletely understood.

Pathological studies demonstrate that RSV primarily infects airway epithelial cells [18]; therefore it is conceivable that a significant component of immune signaling associated with RSV infections originates from airway epithelial responses. Cytokines, chemokines and growth factors are expressed in these cells in response to microbial stimuli [19,20] with numerous pathogen recognition receptors playing a major role in cytokine responses. Pathogen recognition receptors, such as TLRs and retinoic acid–inducible gene-1 (RIG-I)-like receptors [21], induce major signaling cascades following viral stimulation [22]. Indeed, the viral load of RSV correlates with RIG-I mRNA levels [23]. TLR3, −7, −8 and −9 recognize nucleic acid ligands but TLR4 proteins also bind to a major viral antigen of RSV F (fusion) protein [24]. Laboratory of genetics and physiology 2 (LGP2) and melanoma differentiation–associated protein-5 (MDA5) equally could be associated with RSV-induced immune responses [25]. Additionally, ligands for nucleotide-binding oligomerization domain-containing protein (NOD)-like receptors (NLR), such as NOD2, enhance TLR-ligand-induced activation and triggering such signaling cascades are plausible means of developing RSV-specific immunity [26]. Therefore, RSV can induce several pathogen recognition receptors resulting in multiple host immune responses.

In this study we employed *in vitro* and *in vivo* approaches to evaluate the cytokine/chemokine response to RSV infection. Normal human small airway epithelial (SAE) cells and A549 cells were utilized to examine 42 cytokine responses following RSV stimuli. Several newly identified RSV-inducible cytokines (leukemia inhibitory factor (LIF), migration inhibitory factor (MIF), stem cell factor (SCF), CCL27, CXCL12 and stem cell growth factor beta (SCGF-β)) were induced in airways cells *in vitro* and in mouse lungs during a viral infection *in vivo*. The influence of TIR-domain-containing adapter-inducing interferon-β (Trif) and mitochondrial antiviral-signaling protein (MAVS) signaling pathways were examined on RSV-induced cytokine levels in mice and A549 cells. The significance of LIF, an IL-6 cytokine family member, expression during RSV was also determined. LIF is primarily recognized for its ability to preserve the totipotency of embryonic stem cells [27] and is detected in acute respiratory distress syndrome (ARDS) [28] but recently LIF was described to protect the lung from injury during pneumonia [29]. Utilizing neutralizing anti-LIF IgG, LIF was observed to play a critical role in protecting the lung from injury during RSV infection. Our findings indicate that RSV infection induces a significant host cytokine response associated with viral clearance and airway protection.

## Results

### SAE and A549 cells secrete similar cytokines following RSV stimuli

**Figure 1:**
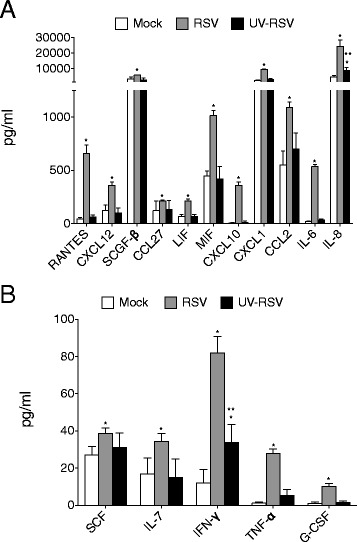
**RSV replication induces host cytokines production in airway epithelial cells.** A549 cells were treated with mock, RSV or UV-inactivated RSV (UV-RSV) for 24 hours. **(A-B)** Secreted cytokines were analyzed by multiplex or ELISA. Graphs are represented as mean cytokine concentration (pg/ml) ± S.E.M, with each measurement performed 3 times on 6 replicates/group. Samples were collected from 3 experiments performed on separate days. * and ** represent a p value less than 0.05 compared to mock or RSV treated cells for each cytokine target, respectively.

A human cytokine array was used to simultaneously detect the levels of 36 different cytokines, chemokines, and acute phase proteins in A549 cells treated with mock and RSV for 24 hours. Protein array analysis identified that a large number of cytokines are released from A549 cells upon RSV stimuli (Additional file 1: Figure S1). The most striking RSV inducible cytokines were IL-6, IL-8, MCP-1/CCL2, MIF, GROα/CXCL1, and RANTES/CCL5 (Additional file 1: Figure S1). To complement the cytokine array analysis, multiplex analysis was performed to examine 42 cytokines, chemokines and growth factors released from A549 cells, in addition to human SAE cells. Both SAE and A549 cells released similar cytokines in response to RSV infection (Table 1). In both A549 and SAE cells, RSV significantly induced IL-1α, IL-1β, IL-1RA, IL-6, IL-7, IL-8, CXCL1, CCL11, fibroblast growth factor (FGF), G-CSF, IFN-γ, CXCL10, CCL2, MIF, platelet-derived growth factor (PDGF-BB), TNF-α, VEGF, LIF, CCL27, CXCL12, SCGF-β and SCF release into media. Interestingly, SAE cells also released IL-16, IL-18, CXCL9, CCL7 and HGF upon RSV stimulation. A549 cells differed to SAE cells in releasing IL-10, IL-12(p70), GM-CSF, CCL3, CCL4 and TNF-related apoptosis-inducing ligand (TRAIL) upon RSV stimulation. RSV-inducible cytokines were examined following stimulation with UV-inactivated RSV in A549 cells, to determine if cytokines are induced following viral detection or viral replication. RANTES/CCL5, CXCL12, SCGF-β, CCL27, LIF, MIF, CXCL10, CXCL1, CCL2, IL-6, SCF, IL-7, TNF-α and G-CSF were all secreted following stimuli with live RSV only (Figure 1). IL-8 and IFN- γ was secreted at significantly higher levels from UV-RSV treated cells compared to mock treated cells but at lower levels to live RSV (Figure 1A). Therefore, RSV replication induces a plethora of immune responses in airway epithelial cells.

**Table 1:** **RSV induces cytokine release from human airway epithelial cells**

	**Airway epithelial cell type**			
	**SAE cells (Type I)**		**A549 (Type II)**	
**Cytokines (pg/ml media)**	**Mock**	**RSV**	**Mock**	**RSV**
**IL-1α**	12.66 ± 1.76	31.75 ± 1.48*	13.52 ± 0.80	34.33 ± 4.3*
**IL-1β**	9.55 ± 1.17	15.50 ± 2.45*	1.28 ± 0.05	2.28 ± 0.45*
**IL1-RA**	40.37 ± 6.37	63.25 ± 4.82*	5.20 ± 0.37	23.28 ± 3.88*
**IL-2**	0.98 ± 0.28	1.44 ± 0.42	0.20 ± 0.05	0.25 ± 0.07
**IL-2RA**	41.65 ± 3.57	44.67 ± 2.12	36.72 ± 0.58	34.53 ± 0.55
**IL-3**	102.7 ± 8.85	100.2 ± 7.37	59.09 ± 3.54	58.08 ± 4.56
**IL-6**	2349 ± 234.6	3260 ± 153.8*	17.21 ± 0.79	534.4 ± 19.22*
**IL-7**	29.36 ± 4.87	55.37 ± 8.32*	17.00 ± 4.57	34.44 ± 4.15*
**IL-8/CXCL8**	2479 ± 154.4	5094 ± 363.5*	142.2 ± 20.70	1203 ± 83.75*
**IL-10**	6.89 ± 1.31	8.13 ± 0.15	5.85 ± 0.27	7.02 ± 0.24*
**IL-12(p40)**	193.4 ± 17.18	183.9 ± 19.78	127.2 ± 10.11	135.0 ± 19.54
**IL-12(p70)**	36.76 ± 5.48	36.09 ± 5.94	14.54 ± 1.29	19.86 ± 0.81*
**IL-16**	115.7 ± 14.5	223.5 ± 19.04*	652.1 ± 32.79	387.5 ± 45.97
**IL-18**	6.70 ± 0.71	11.94 ± 0.92*	6.24 ± 0.31	7.06 ± 0.37
**GROα/CXCL1**	8482 ± 428	11119 ± 639*	2431 ± 177.7	9473 ± 366.4*
**Eotaxin/CCL11**	5.16 ± 0.24	10.37 ± 0.62*	1.38 ± 0.16	3.43 ± 0.37*
**FGF**	6.60 ± 0.30	10.72 ± 1.41*	4.24 ± 0.51	13.92 ± 2.16*
**G-CSF/CSF-3**	945.6 ± 72.01	**1403 ± 130.2**	1.00 ± 0.79	10.16 ± 1.57*
**GM-CSF/CSF-2**	9.74 ± 2.99	10.04 ± 3.15	3.73 ± 0.44	7.12 ± 0.51*
**M-CSF/CSF-1**	73.03 ± 8.60	98.50 ± 6.41	2.90 ± 0.74	4.55 ± 0.63
**IFN-α2**	4.62 ± 0.32	4.97 ± 0.27	4.46 ± 0.04	4.55 ± 0.76
**IFN-γ**	95.39 ± 13.08	171.6 ± 12.04*	12.38 ± 1.98	81.81 ± 8.88*
**MIG/CXCL9**	29.01 ± 3.91	61.41 ± 2.99*	53.83 ± 5.43	104.81 ± 12.11
**IP10/CXCL10**	337.6 ± 93.99	10580 ± 546*	5.13 ± 0.35	1836 ± 97.5*
**MCP-1/CCL2**	146.2 ± 15.51	266.0 ± 42.71*	557.1 ± 30.48	1088 ± 56.54*
**MCP-3/CCL7**	71.68 ± 8.97	215.8 ± 17.44*	96.03 ± 10.57	78.41 ± 11.14
**MIP-1α/CCL3**	1.88 ± 0.12	2.38 ± 0.36	1.25 ± 0.09	16.96 ± 0.42*
**MIP-1β/CCL4**	1.55 ± 0.32	2.75 ± 0.45	0.77 ± 0.13	89.04 ± 7.52*
**MIF**	2123 ± 186.2	7435 ± 179.5*	454.0 ± 20.8	1015 ± 47.5*
**PDGF-BB**	6.91 ± 1.01	16.90 ± 2.98*	3.68 ± 0.59	10.23 ± 0.16*
**RANTES/CCL5**	206.8 ± 10.32	339.9 ± 29.54*	44.30 ± 8.53	656.9 ± 82.5*
**TNF-α**	16.78 ± 0.57	32.40 ± 3.27*	1.14 ± 0.12	27.83 ± 2.37*
**TNF-β/LTA**	21.98 ± 1.39	21.09 ± 0.78	17.59 ± 0.18	17.41 ± 0.72
**VEGF**	3717 ± 372.2	5256 ± 304.3*	1585 ± 108.9	3297 ± 146.2*
**TRAIL**	135.7 ± 13.64	209.2 ± 6.82	151.4 ± 10.16	191.9 ± 5.06*
**β-NGF**	23.92 ± 1.76	23.81 ± 2.23	20.50 ± 1.17	24.12 ± 2.39
**LIF**	114.9 ± 11.92	227.4 ± 6.7*	64.70 ± 2.41	212.7 ± 15.88*
**HGF**	63.33 ± 4.98	81.84 ± 1.41*	71.23 ± 1.47	69.34 ± 1.71
**CTAK/ CCL27**	130.5 ± 9.87	249.3 ± 9.73*	122.5 ± 2.40	211.7 ± 8.91*
**SDF-1α/CXCL12**	672.0 ± 31.7	1504 ± 97.33*	121.6 ± 9.61	359.4 ± 28.6*
**SCGF-**β	685.7 ± 32.1	1000 ± 73.1*	3575 ± 191.2	5905 ± 161.9*
**SCF**	19.09 ± 1.08	42.87 ± 0.18*	27.50 ± 1.52	38.66 ± 3.03*

Cytokine levels were determined in cell culture supernatants from A549 or SAE cells 24 hours post RSV challenge, by multiplex analysis. Values are represented as mean media concentration (pg/ml) ± S.E.M. Assays were performed in triplicate from samples collected on separate days, where n = 10 samples/group. Bold numbers denoted by *represents a p value less than 0.05 compared to mock treated cells.

### MIF, LIF, CCL27, CXCL12/SDF-1α, SCGF-β and SCF are expressed in the airways of mice following RSV challenge

**Figure 2:**
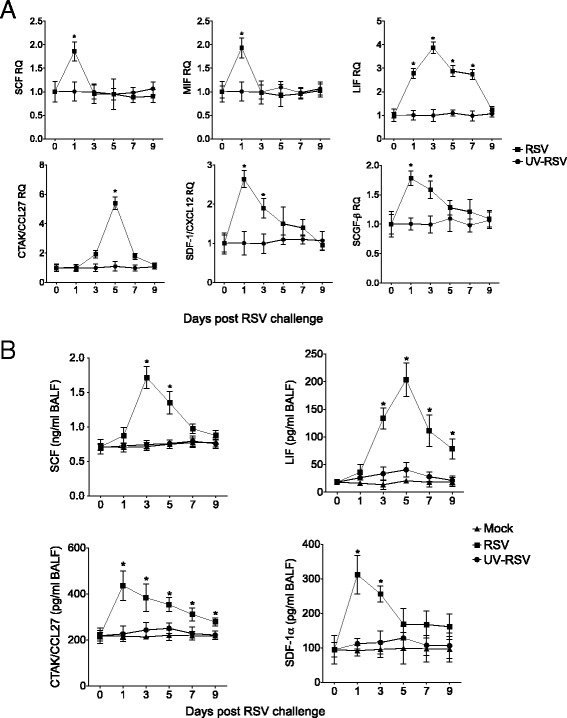
**RSV challenge induces MIF, LIF, CCL27, CXCL12, SCGF-β and SCF airway expression.** FVB/NJ mice were infected with 1x10^6^ pfu of RSV, UV-RSV or mock and animals were euthanized on days 0, 1, 3, 5, 7 and 9-post challenge. MIF, LIF, CTAK/CCL27, SDF-1/CXCL12, SCGF-β and SCF **(A)** lung gene expression and **(B)** BALF protein levels were analyzed by qPCR and multiplex, respectively. Graphs are represented as relative quantification (RQ) or BALF concentration of the mean ± S.E.M, with each measurement performed 3 times on 10 animals/group. *Represents a p value less than 0.05 compared to mock treated mice on each corresponding day.

Gene expression and BALF protein levels for MIF, LIF, CCL27, CXCL12/SDF-1α, SCGF-β and SCF were examined in wild-type (FVB/NJ) mice 0, 1, 3, 5, 7, and 9 days post mock, RSV or UV-RSV intranasal challenge (Figure 2), as these cytokines have not been reported in the literature to be RSV inducible. Increased gene expression of MIF, LIF, CXCL12, SCGF-β and SCF was observed following 1 day of RSV challenge (Figure 2A). RSV inducible CCL27 was only observed 5 days post challenge. UV-inactivated RSV did not alter airway expression of these cytokines (Figure 2A). Multiplex or ELISA was performed to examine BALF protein levels of LIF, CCL27, CXCL12/SDF-1α, and SCF. Similarly to gene expression profiles, BALF levels of SCF, LIF, CCL27/CTAK and CXCL12/SDF-1α were all enhanced in mice exposed to RSV infection compared to mock or UV-RSV treated animals (Figure 2B). These new RSV-inducible targets represent new plausible targets for investigation in the human disease.

### RSV activates TLRs and RIG-I-like receptors (RLR)

**Figure 3:**
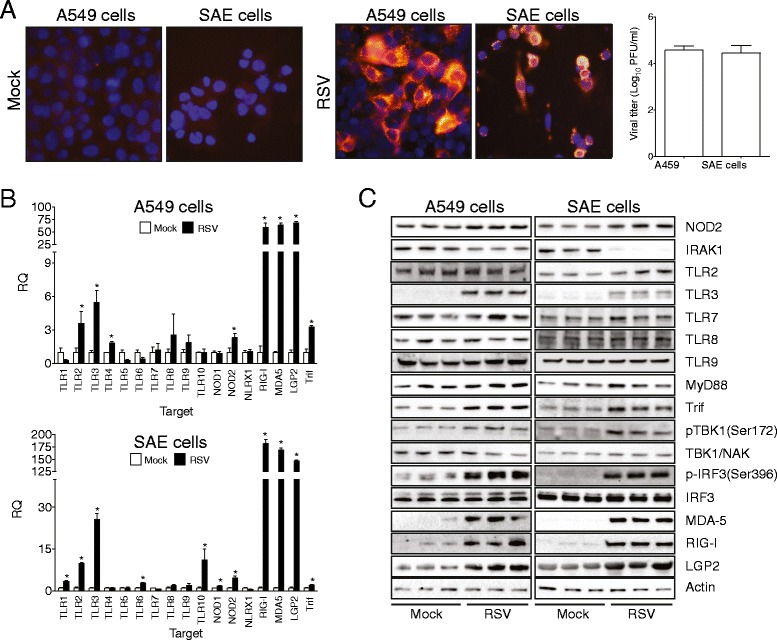
**RSV induces multiple pathogen recognition receptors in A549 and SAE cells. (A)** Representative immunofluorescence staining of SAE and A549 cells treated with mock or RSV and stained 24 hours later with RSV F-specific monoclonal antibody. Cell lysates were examined for viable viruses by plaque assays. **(B)** qPCR and **(C)** immunblots were performed to examine levels of pathogen recognition receptors and downstream signaling in cells 1-day post RSV infection. Graphs are represented as **(B)** PFU (Log_10_) or **(C)** RQ of the mean ± S.E.M, where n = 10 replicates/group and each assay was performed in triplicate. Each experiment was performed on samples obtained from 3 experiments from separate days. *Represents a p value less than 0.05 compared to mock treated mice.

Nasopharyngeal cells from infants with RSV infection have gene expression differences for MDA-5, RIG-1, TLR-7, and TLR-8 [23]. Since RSV infection induces a large repertoire of host cytokines, chemokines and growth factors, several of the major pathogen recognition receptors were profiled in each cell type to determine the potential regulators of the immune response to RSV infection. Therefore, qPCR and immunoblots were performed for p-IRF3(ser396), IRF3, p-TBK1(ser172), TBK-1/NAK, RIG-I, LGP2, MDA5, MAVS, LGP2, TLR3, TLR7, TLR8, TLR9, Trif, MyD88, IRAK1, NOD2 and β-actin. The remaining TLR genes and other NOD genes (NOD1 and NLXR1) were also profiled in both cell types. A549 and SAE cells became readily infected with RSV 24 hours after incubation (Figure 3A) and had significant increases in RIG-I, MDA5, LGP2, NOD2, Trif, TLR2 and TLR3 gene expression in both cell types (Figure 3B). TLR1, 6 and 10 gene expressions were also enhanced in SAE cells following RSV infection. TLR4 gene expression was increased in A549s cells. Protein profiles were analyzed for several of the receptors with the greatest gene induction with RSV infection, by immunoblots. Increased cellular protein levels of RIG-I, MDA5, LGP2, Trif and TLR3 were observed by immunblotting in both A549 and SAE cells (Figure 3C). Utilizing densitometry analysis, no significant changes in NOD2 and TLR2 protein levels were observed in either A549 or SAE cells possibility due to lower induction of gene expression for these targets (Additional file 1: Figure S2). To investigate the activation status of receptors, downstream signaling transduction analysis was performed for the phosphorylation of IRF3, TBK1, degradation of IRAK1 and increased expression of Trif and MyD88. IRF3 and TBK1 underwent phosphorylation following RSV infection, confirming activation of these pathways (Figure 3C and Additional file 1: Figure S2). Equally, increases in Trif and decreased IRAK1 levels were observed after RSV challenge (Figure 3C and Additional file 1: Figure S2), further confirming that several receptors underwent activation. Due to the activation intensity of the RLR, activation of IRF3 and induction of the Trif/TLR3, *in vivo* studies were undertaken specifically examining the Trif and RLR pathways.

### *Trif* and *Mavs* KO mice have reduced cytokine responses to RSV challenge

**Figure 4:**
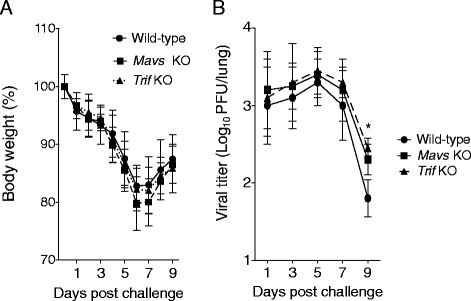
**Loss of** ***Trif*** **or** ***Mavs*** **expression enhances RSV viral titer in the lungs of mice. (A)** RSV infection resulted in a drop in body weight in all animal groups and **(B)** a significant increase in the viral load 9 days post RSV challenge in the *Mavs* KO mouse group compared to mock treated mice. Graphs are represented as mean ± S.E.M., where each measurement was performed 3 times on 10 animals/group. *Represents a p value less than 0.05 comparing *Mavs* KO mice to wild-type mice 9 days post RSV challenge.

Others have reported that RIG-I is essential for host immune defenses against RSV [30,31] and our *in vitro* studies suggest that both TLR3 and RLR signaling are highly responsive to RSV. To assess the importance of RSV-induced TLR3 and RLR signaling on cytokine production, wild-type (FVB/NJ), *Trif* and *Mavs* KO mice were infected with RSV and cytokines levels were investigated by qPCR on lung tissue and multiplex analysis on BALF. *Mavs* KO mice were utilized as MAVS links RIG-I and MDA5 to antiviral effectors responses. Mice were exposed to RSV and euthanized days 1, 3, 5, 7 and 9 days after the RSV challenge. Loss of *Trif* and *Mavs* expression in mice did not significantly alter loss in body weight following RSV infection compared to wild-type mice (Figure 4A) but loss of *Mavs* or *Trif* resulted in a greater viral load in the airway tissue on day 9 following infection compared to wild-type mice (Figure 4B).

Cytokine release into the airways was assessed by multiplex analysis 3 days post RSV challenge and RSV infection yielded a significant release of IL-1α, IL-1β, IL-2, IL-4, IL-5, IL-6, IL-12(p40), IL-12(p70), IL-13, IL-15, IFN-γ, MCP-1/CCL2, CCL5, CCL3, CCL4, CCL11, LIF, CTAK/CCL27, SDF-1α/CXCL12, SCF and CXCL1 into the BALF of control mice (Table 2). RSV also increased gene expression of IL-27, CXCL9, CXCL10, CXCL11, MIF and SCGF-β in the airways of control mice 1-day post RSV challenge (Table 3). Loss of *Mavs* expression subdued RSV-induced IL-1β, IL-4, IL-5, IL-6, IL-12(p40), IFN-γ, CCL2, CCL3, CCL5 and CXCL1 release into the airways (Table 2). *Mavs* KO mice also had significantly reduced IL-22, CXCL9, CXCL10 and MIF gene expression compared to wild-type mice (Table 3). Loss of *Trif* expression altered the RSV induced release of IL-1β, IL-5, CXCL12 and IFN-γ (Table 2) and airway gene expression of MIF, LIF and CXCL12 (Table 2).

**Table 2:** **RSV infections induce airway cytokine release in mice**

	**Wild-type**		***Trif*** **KO**		***Mavs*** **KO**	
**Cytokine (pg/ml BALF)**	**Mock**	**RSV**	**Mock**	**RSV**	**Mock**	**RSV**
**IL-1α**	3.54 ± 1.51	30.11 ± 9.64*	9.62 ± 8.57	25.53 ± 5.33	6.74 ± 3.56	24.55 ± 3.36
**IL-1β**	66.61 ± 5.52	78.55 ± 6.66*	67.05 ± 6.19	66.16 ± 5.00*	56.88 ± 2.83	**49.18 ± 4.06** ^**#**^
**IL-2**	7.25 ± 0.32	5.52 ± 0.31*	6.44 ± 0.03	5.91 ± 1.22	5.36 ± 0.35	6.91 ± 0.75
**IL-3**	29.39 ± 1.54	28.87 ± 0.84	30.50 ± 1.55	28.95 ± 1.47	22.98 ± 2.89	24.37 ± 0.51
**IL-4**	15.00 ± 0.01	48.12 ± 0.02*	17.21 ± 0.01	32.89 ± 0.01	14.41 ± 0.01	**16.33 ± 0.01** ^**#**^
**IL-5**	4.15 ± 0.27	20.00 ± 5.42*	5.39 ± 0.63	**7.55 ± 1.46** ^**#**^	4.37 ± 0.15	**4.81 ± 0.50** ^**#**^
**IL-6**	3.78 ± 0.44	9.63 ± 3.80*	4.19 ± 0.57	9.79 ± 3.16	3.18 ± 0.31	**5.55 ± 1.16** ^**#**^
**IL-9**	204.10 ± 11.28	233.11 ± 18.25	230.31 ± 37.74	216.01 ± 18.58	181.42 ± 12.58	228.93 ± 19.01
**IL-10**	61.43 ± 9.92	76.45 ± 22.60	46.08 ± 8.18	166.2 ± 55.5	51.13 ± 8.32	63.33 ± 12.23
**IL-12(p40)**	8.69 ± 0.17	11.95 ± 1.65*	9.95 ± 0.97	10.99 ± 1.18	6.89 ± 1.06	**8.74 ± 0.28** ^**#**^
**IL-12(p70)**	11.34 ± 0.98	10.23 ± 1.43	13.14 ± 3.60	14.89 ± 2.85	8.60 ± 2.10	12.01 ± 3.70
**IL-13**	48.41 ± 2.44	29.61 ± 3.50*	41.84 ± 4.94	26.51 ± 2.15	47.99 ± 2.33	30.31 ± 6.52
**IL-15**	6.96 ± 1.09	14.25 ± 3.31*	13.46 ± 3.62	9.83 ± 1.89	7.42 ± 2.18	10.83 ± 1.68
**IL-17**	43.66 ± 5.10	64.17 ± 16.72	37.61 ± 30.20	79.81 ± 13.48	41.34 ± 5.01	55.44 ± 4.68
**IFN-γ**	12.15 ± 0.76	35.47 ± 11.26*	10.19 ± 1.26	**8.24 ± 0.61** ^**#**^	11.74 ± 5.04	**13.22 ± 3.78** ^**#**^
**MCP-1/CCL2**	64.57 ± 2.12	85.14 ± 8.23*	67.87 ± 5.15	81.61 ± 7.86	63.22 ± 5.01	**69.99 ± 4.82** ^**#**^
**RANTES/CCL5**	12.41 ± 0.84	107.32 ± 21.70*	18.02 ± 3.32	128.81 ± 23.27	15.01 ± 0.98	**68.32 ± 11.90** ^**#**^
**TNF-α**	524.98 ± 41.34	438.88 ± 55.48	602.71 ± 38.15	460.01 ± 84.64	504.18 ± 32.14	448.88 ± 34.48
**MIP-1α/CCL3**	105.01 ± 1.38	164.72 ± 5.56*	123.02 ± 18.29	132.58 ± 12.77	95.96 ± 4.84	**106.61 ± 4.60** ^**#**^
**MIP-1β/CCL4**	66.37 ± 2.89	112.91 ± 19.50*	49.59 ± 4.74	103.10 ± 18.43	54.40 ± 10.08	127.45 ± 41.05
**MIP-2/CXCL2**	10.22 ± 0.56	10.82 ± 2.04	14.86 ± 1.86	13.40 ± 2.47	9.15 ± 0.80	13.00 ± 1.78
**Eotaxin/CCL11**	5.72 ± 0.55	25.02 ± 5.97*	9.26 ± 2.68	15.37 ± 3.80	5.94 ± 0.50	13.62 ± 3.08
**GM-CSF/CSF2**	41.63 ± 3.79	45.57 ± 3.25	49.61 ± 3.60	57.88 ± 6.41	37.14 ± 6.15	45.93 ± 2.41
**M-CSF/CSF1**	24.93 ± 2.08	26.73 ± 2.47	29.13 ± 2.97	28.85 ± 3.79	20.40 ± 1.97	25.94 ± 2.84
**LIF**	12.37 ± 5.4	70.18 ± 12.33*	11.12 ± 2.12	60.42 ± 10.32	12.91 ± 3.99	72.32 ± 7.69
**CTAK/CCL27**	231 ± 21.23	430 ± 43.21*	220 ± 18.91	389.1 ± 21.98	198.3 ± 10.99	424.7 ± 34.76
**SDF-1α/CXCL12**	94.0 ± 10.16	312.2 ± 50.96*	51.1 ± 23.23	**77.21 ± 13.11** ^**#**^	110.21 ± 20.20	296.19 ± 15.07
**SCF**	711.00 ± 40.15	1713.06 ± 98.19*	721.79 ± 88.95	1392.90 ± 99.41	702.83 ± 76.41	1721.22 ± 70.60
**GROα/KC/CXCL1**	1.02 ± 0.45	**81.35 ± 16.70**	1.24 ± 0.16	81.29 ± 23.27	4.35 ± 0.97	**48.55 ± 20.84** ^**#**^

Cytokine levels were determined in BALF from wild-type, *Trif* KO and *Mavs* KO mice 3 days post RSV challenge, by multiplex analysis. Values are represented as mean BALF concentration (pg/ml) ± S.E.M. Each assay was performed in triplicate, where n = 10 animals/group. Bold numbers denoted by *represents a p value less than 0.05 compared to mock treated mice. ^#^denotes a p value less than 0.05 compared to RSV treated wild-type mice.

**Table 3:** **RSV infections enhance airway cytokine gene expression**

	**Wild-type**		***Trif*** **KO**		***Mavs*** **KO**	
**Cytokine (pg/ml)**	**Mock**	**RSV**	**Mock**	**RSV**	**Mock**	**RSV**
IL-4	1.00 ± 0.40	4.67 ± 1.26*	1.26 ± 0.33	3.97 ± 0.53	0.27 ± 0.14	**0.23 ± 0.10** ^**#**^
IL-6	1.00 ± 0.10	4.15 ± 0.14*	1.30 ± 0.33	3.32 ± 0.52	1.81 ± 0.71	**1.61 ± 0.57** ^**#**^
IL-18	1.00 ± 0.19	0.71 ± 0.16	0.64 ± 0.15	0.43 ± 0.03	0.51 ± 0.06	0.60 ± 0.34
IL-22	1.00 ± 0.41	1.19 ± 0.16	0.98 ± 0.07	1.49 ± 0.49	0.54 ± 0.30	**0.45 ± 0.08** ^**#**^
IL-23	1.00 ± 0.41	1.48 ± 0.27	0.75 ± 0.17	1.41 ± 0.20	0.19 ± 0.15	1.03 ± 0.02
IL-27	1.00 ± 0.15	2.00 ± 0.10*	0.98 ± 0.31	1.88 ± 0.39	0.49 ± 0.06	1.63 ± 0.09
CXCL1/KC	1.00 ± 0.12	11.91 ± 0.05*	1.56 ± 0.67	12.04 ± 0.37	0.29 ± 0.11	**3.23 ± 0.12** ^**#**^
MIG/CXCL9	1.00 ± 0.58	160.81 ± 17.67*	3.18 ± 1.10	148.83 ± 16.68	0.23 ± 0.14	**29.47 ± 5.42** ^**#**^
IP10/CXCL10	1.00 ± 0.18	8.07 ± 1.62*	1.78 ± 0.43	9.77 ± 2.51	0.312 ± 0.10	**3.59 ± 2.07** ^**#**^
IP9/CXCL11	1.00 ± 0.12	2.61 ± 0.26*	1.30 ± 0.44	4.38 ± 0.85	0.18 ± 0.16	3.19 ± 1.05
MIF	1.00 ± 0.19	2.40 ± 0.26*	1.09 ± 0.15	**0.90 ± 0.41** ^**#**^	0.93 ± 0.11	**0.52 ± 0.40** ^**#**^
LIF	1.00 ± 0.14	2.71 ± 0.09*	1.16 ± 0.66	**1.01 ± 0.27** ^**#**^	0.99 ± 0.11	2.43 ± 0.13
CTAK/CCL27	1.00 ± 0.28	5.79 ± 0.67*	1.18 ± 0.18	4.23 ± 0.68	0.73 ± 0.18	4.27 ± 0.42
SDF-1α/CXCL12	1.00 ± 0.16	2.67 ± 0.96*	1.28 ± 0.23	**1.57 ± 0.51** ^**#**^	0.51 ± 0.20	2.59 ± 1.07
SCGF-β	1.00 ± 0.22	1.87 ± 0.16*	1.33 ± 0.14	1.88 ± 0.81	0.88 ± 0.26	1.19 ± 0.15
SCF	1.00 ± 0.15	1.60 ± 0.16*	1.09 ± 0.25	1.20 ± 0.41	0.93 ± 0.11	1.82 ± 0.40
TGF-β	1.00 ± 0.16	1.47 ± 0.06	0.84 ± 0.19	1.00 ± 0.43	0.51 ± 0.01	0.62 ± 0.43
MCP-1/CCL2	1.00 ± 0.08	12.39 ± 3.01*	2.38 ± 0.73	20.45 ± 4.31	0.45 ± 0.07	**5.13 ± 1.33** ^**#**^
MIP-1α/CCL3	1.00 ± 0.08	10.17 ± 1.51*	1.68 ± 0.40	13.64 ± 1.51	0.58 ± 0.14	**3.37 ± 0.83** ^**#**^

Cytokine gene expression levels were determined in lung tissue from wild-type, *Trif* KO and *Mavs* KO mice 1 days post RSV challenge by qPCR, except CTAK/CCL27 which was examined 5 days post RSV challenge. Values are represented as mean RQ (relative to mock treated animals, using β-Actin as an endogenous control.) ± S.E.M. Each assay was performed in triplicate, where n = 10 animals/group. Bold numbers denoted by *represents a p value less than 0.05 compared to mock treated mice. ^#^denotes a p value less than 0.05 compared to RSV treated wild-type mice.

### Silencing RIG-I expression in A549 cells subdues cytokine production

**Figure 5:**
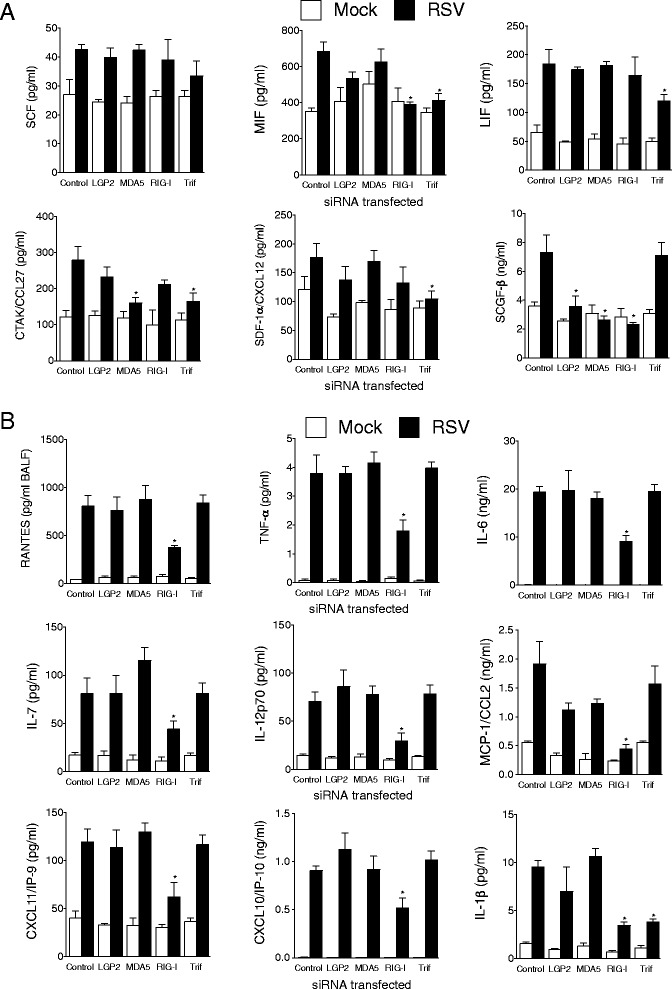
**Silencing RIG-I subdues RSV induced cytokine release from A549 cells. (A-B)** Multiplex or ELISA analysis was performed on secreted cytokines from A549 cells transfected with siRNA targeting RIG-I (DDX58), LGP2 (DHX58), MDA5 (IFIH1), Trif or a negative sequence control. Graphs are represented as mean cytokine concentration ± S.E.M, where n = 10 replicates/group and each assay were performed in triplicate. Each experiment was performed on samples obtained from 3 experiments from separate days. *Represents a p value less than 0.05 compared to control siRNA treated cells following RSV infection.

RLRs (RIG-I, MDA5 and LGP2) and Trif were silenced in A549 cells to establish which RLR signaling was required for RSV inducible host cytokines (See Additional file 1: Figure S3 for RLR and Trif knockdown). Loss of RIG-I expression significantly decreased RSV induced IL-1β, IL-6, IL-7, IL-12(p70), MCP-1/CCL2, IP-9/CXCL11, IP-10/CXCL10, TNF-α, MIF, RANTES/CCL5 and SCGF-β (Figure 5). Interestingly, SCGF-β release was altered following loss of expression of RIG-I, MDA5 and LGP2 (Figure 5A). CTAK/CCL27 release was only altered following silencing of MDA5 or Trif expression (Figure 5A). Trif expression regulated MIF, LIF, CTAK/CCL27, SDF-1α/CXCL12 and IL-1β (Figure 5). Therefore, these results show that host RIG-I is the primary RLR to regulate RSV-mediated immune responses.

### Neutralization LIF enhances lung injury during an RSV infection

**Figure 6:**
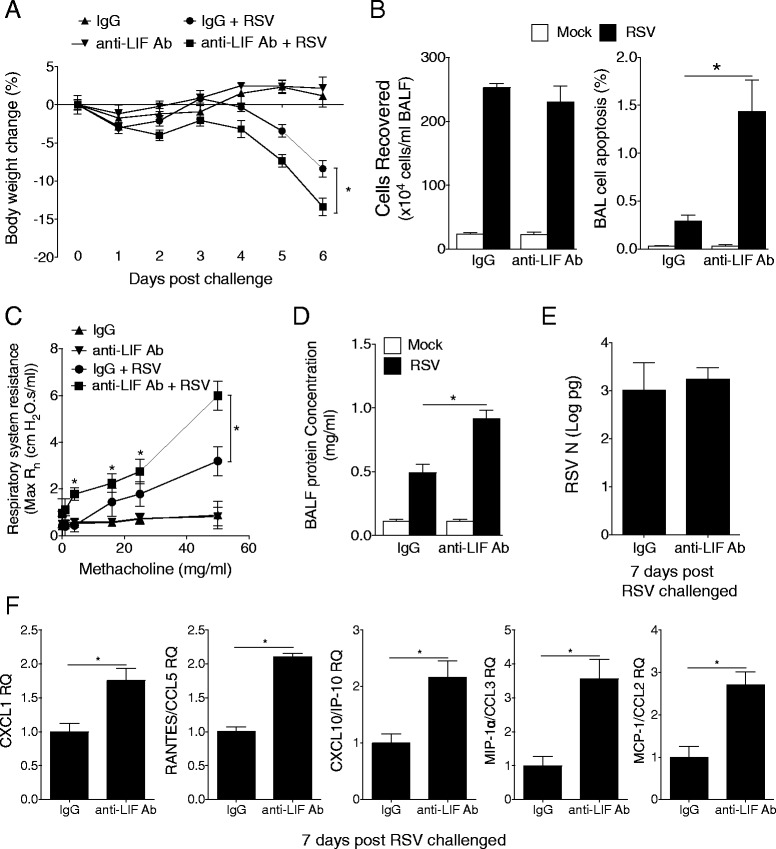
**RSV induced airway injury is enhanced following LIF neutralization.** FVB/NJ mice were IP injected with control IgG or anti-LIF IgG prior to intranasal infected with 1x10^6^ pfu of RSV and animals were euthanized 7-days post infection. **(A)** Animal body weight, **(B)** BALF cellularity and BALF cells undergoing apoptosis were determined. **(C)** Airway hyperresponsiveness to increasing doses of methacholine was assessed in each animal group. **(D)** RSV infected animals receiving anti-LIF IgG have enhanced protein content in BALF compared to mice administered control IgG. **(E)** Neutralizing LIF levels did not impact on RSV viral multiplicity, demonstrated by qPCR for RSV N. **(F)** CXCL1, RANTES, CCL5, CXCL10/IP-10, MIP-1α/CCL3 and MCP-1/CCL2 gene expression in lung tissue was analyzed by qPCR. Graphs are represented as mean ± S.E.M., where each measurement was performed 3 times on 10 animals/group. * represents a p value less than 0.05 comparing control IgG and anti-LIF IgG treated mice both infected with RSV.

Administration of recombinant LIF to the airways has a protective effect in response to LPS treatment [32], hyperoxia [33] and to bacterial pneumonia [29]. The functional relevance of LIF expression during a RSV infection is unknown. Administration of anti-LIF IgG resulted in RSV-infected animals losing weight faster than control IgG treated littermates (Figure 6A). The total number of immune cells in the BALF was comparable between RSV treated groups (Figure 6B). However, BALF cells in the animals administered anti-LIF IgG were undergoing apoptosis at a greater rate than control IgG animals (Figure 6B). Inhibition of LIF signaling also subdued RSV induced airway hyperresponsiveness, demonstrated by respiratory system resistance (Rn) measurements during a methacholine dose challenge (Figure 6C). Anti-LIF IgG treated mice had significantly higher protein concentration (Figure 6D) in the BALF compared to infected control IgG treated mice, suggesting potentially increased damage and disruption of the epithelial cell barrier. Lung RSV burdens were not significantly affected by LIF blockade seven days following RSV challenge (Figure 6E). Lung cytokine gene expression was determined by qPCR and blocking LIF signaling resulted in enhanced CXCL1, RANTES/CCL5, CXCL10/IP-10, MIP-1α/CCL3 and MCP-1/CCL2 compared to control IgG treated mice, 7-days post RSV infection (Figure 6F). Altogether, these results suggest that LIF expression suppresses lung cell apoptosis, airway hyperresponsiveness, epithelial cell barrier damage, cytokine production and subsequently airway injury.

## Discussion

To our knowledge, these findings represent the first evidence that MIF, LIF, CTAK/CCL27, SDF-1α/CXCL12, SCGF-β and SCF are expressed during an RSV infection and LIF signaling is critical to prevent lung damage. Others and we demonstrate that a spectrum of cytokines is released to counter RSV infection [1,7-10,34] but further analysis is required to determine the role of these immune responses in RSV pathology. The results of this study demonstrate that RLRs and Trif signaling pathways play major roles in the immune response to RSV infection for both inflammation induction and resolution. Interestingly, 34 of the 42 cytokines examined in A549 and SAE cells were released upon RSV stimulation. This study highlights the robust epithelial responses to RSV infection, characterizes two major signaling cascade needed to counter viral infectivity and identified the role of LIF signaling during an RSV infection.

Many of the RSV-inducible cytokines described in this study have already been described in clinical samples, *in vitro* epithelial cell culture or in animals models [1,7-16], such as elevated MIG/CXCL9 levels in BALB/c mice inoculated with RSV [35]. Importantly, increases in media or mouse BALF IL1-RA, IL-6, IL-7, CXCL10/IP-10, CXCL8/IL-8, CCL2/MCP-1, CCL3/MIP-1α, CCL4/ MIP-1β, TNF-α, IFN-γ and CCL5/RANTES following RSV infection reflects similar observations from clinical studies [10,36]. To our knowledge, we are the first to identify that RSV infection in epithelial cells and mice can induce MIF, LIF, CTAK/CCL27, SDF-1α/CXCL12, SCGF-β and SCF expression and secretions. This is an important finding as each new target can potentially play a different role in RSV clearance. MIF activates lymphocytes, granulocytes and monocytes/macrophages and plays a key role in several host immune cell responses [37]. CTAK/CCL27 is a cytokine associated with T cell activation and migration [38] and is elevated in severe tuberculosis cases [39]. Recombinant protein SDF-1α/CXCL12 does not prevent RSV infection of Hep2 cells [40] but SDF-1α/CXCL12 is strongly chemotactic for lymphocytes [41] and contributes to pulmonary fibrosis [42]. The potential role of SCGF-β in RSV infections is not as straightforward to elucidate but SCGF-β can support growth of primitive hematopoietic cells [43] and can promotes proliferation of erythroid or myeloid progenitors. SCF and its soluble receptor c-kit correlate with asthma severity [44]. This study identified an important role for LIF in RSV infection. Therefore, identifying these new RSV inducible targets in clinical samples and their role in viral clearance may provide important insights into host responses to RSV infection.

Other investigators have demonstrated the therapeutic potential of LIF expression in other models of lung injury, as it protects against lung injury [29,32,33]. Recombinant LIF reduces cytokine expression [29] and alters alveolar neutrophil numbers [29,32]. *Lif* KO animals have increased expression of CXCL1, GM-CSF, RANTES/CCL5 and MIP-1α/CCL3 early during an experimental autoimmune encephalomyelitis model and reduced CCL2, CCL3, and CXCL10 at a later stage of the disease [45]. In this study neutralizing LIF signaling enhanced CXCL1, RANTES/CCL5, CXCL10/IP-10, MIP-1α/CCL3 and MCP-1/CCL2 at day 7 of an RSV infection. Therefore LIF expression has the potential to regulate key factors in the immune system. Targeted overexpression of LIF in mouse airway epithelial cells significantly protects the airways during hyperoxia, with improved survival and decreased pulmonary edema [33]. Other IL-6 family members (IL-6 [46] and IL-11 [47]) have similar functions, which suggest that IL-6 family cytokines may collectively protect lungs from injury. LIF is a prominent STAT3-activating cytokine that facilitates tissue protection during pneumonia [29] and plays a similar airway protective role during RSV infection. The significance of LIF expression and STAT3 activation in RSV infection represents an intriguing area for further study. LIF has been shown to regulate apoptosis with some investigators suggesting LIF acts as a pro-apoptotic mediator [48,49] while others have demonstrated LIF to have anti-apoptotic potential [50,51]. In this study increased apoptotic BAL cells were observed in animals administered anti-LIF IgG prior to RSV infection. Enhanced Fas, Fap, Il24, and Tnfsf15 expression is observed following LIF depletion in animals with bacterial pneumonia [29], which could contribute to the increased apoptosis observed here. Whether this induction of apoptosis contributes to the pathology observed in this study is unknown and requires further investigation. Epithelial damage correlates with airway hyperreactivity in asthma patients [52] and the increased protein levels in the BALF of animals treated with anti-LIF IgG prior to RSV infection could also contribute to the enhanced airway resistance observed in these animals. LIF enhances the maintenance of stem cells [27], which may directly impact lung tissue regeneration and repair [53], similar to IL-6 activation of STAT3 [54]. These data reveal the vast biological processes of LIF signaling during RSV infection. Whether the other cytokines identified in this study also play such major roles on disease pathology needs to be addressed.

Our study also reveals that Trif and RLR signaling regulate a significant proportion of the cytokines induced by RSV infection, with LIF expression sensitive to Trif activation. Prior to undertaking this study, several pathogen recognition receptors were associated with regulating the immune response against RSV infection, with RIG-I [55], TLR7 [56] and TLR3 [57] known to affect numerous cytokine responses. Several investigators characterized the RIG-I-like family proteins as critical for the detection of RSV, using human cell lines [30] and knockout mice [58]. RIG-I principally recognizes dsRNA [59]; however Sendai virus defective interfering RNA [60] and the genomic “panhandle” structure of influenza virus [61] activate RLR signaling. These “panhandle” structures allow negative-strand RNA viral genomes to achieve partially double-stranded RNA [62], which may allow RSV to induce RLRs. Our study observed early cytokine responses following RSV infection and identified a pronounced RIG-I response, which could be lost over time following NS1 or NS2 binding to RIG-I/MDA5 as reported by others [63,64]. Also, RSV induction of TLR3 is regulated by RIG-I-dependent IFN-β secretions from infected epithelial cells, which is mediated by both IFN response-stimulated element (ISRE) and signal transducer and activator of transcription (STAT) sites in its proximal promoter [65]. Therefore there are multiple levels of RLRs regulation in RSV infections that can both trigger RLRs responses or subsequently adapt to block RLR-mediated signaling.

Recently, several investigators have utilized well-defined primary cell culture techniques to investigate viral-host interactions [1,66,67], which may be a closer representation of human RSV infections compared to the approach we employed here. IFN-α/β are not secreted by RSV-infected well-defined bronchial epithelial cells [66], suggesting that there could be major signaling differences in well-defined primary cell culture techniques and monolayers. However, our overall findings suggest that utilizing cell monolayers in combination with mouse models is an acceptable approach as our cytokine profile are comparable to that observed in the human disease state [10]. In addition, our study has exposed new RSV inducible host immune response cytokines. Since we only explored epithelial cell responses *in vitro*, our *in vivo* parallel approach also validates our monolayer results and suggests that the epithelium is a significant source of initial immune responses following RSV infection.

## Conclusions

Our studies demonstrate that RSV exposure stimulates a cascade of early immune response in epithelial cells and a large number of these responses act in an RLR/Trif dependent manner. These findings also identified new cytokines that are triggered by RSV lung infection, with LIF signaling critical for the protection of the lung from injury during RSV infection. Profiling the loss and gain of the other identified cytokines (MIF, CTAK/CCL27, SDF-1α/CXCL12, SCGF-β and SCF) over the course of an RSV infection may help to decipher the relevancy of these cytokines in viral clearance and lung integrity, and represents important questions to be addressed by future studies.

## Methods

### RSV culture

Human RSV strain A2 (ATCC, Manassas, VA; #VR-1540) was infected at a multiplicity of 0.1 into Hep2 cells. The virus was allowed to grow for 5 days at 37°C in a 5% CO_2_ atmosphere. The infected Hep2 monolayers were collected and the virus was released by sonication. Cell debris was removed by centrifugation at 2500 *× g* for 5 minutes at 4°C. Virus was collected by centrifuging the supernatant for 2 hours at 22000 *× g* at 4°C. Virus were suspended in culture media and snap frozen and maintained at −80°C. Infectious virus titers were determined on Hep2 cells by performing serial dilution of the RSV stocks and counting infected cells stained for indirect immunofluorescence with an RSV F-specific monoclonal antibody (Abcam, Cambridge, MA). Additionally, plaque assays were performed as previously described [68] on Hep2 cells using methyl cellulose overlay media and staining with 0.5 mg/ml thiazolyl blue tetrazolium bromide (MTT; Sigma Aldrich) solution for 3 hours at 37°C. Non-infected cells were processed in the same manner as RSV infected cells and the resulting sample collection was used as a mock control. For experiments examining the effects of non-infectious RSV (UV-RSV), RSV preparations were UV-inactivated.

### Cell culture

Monolayers of human SAE cells (Lonza, Walkersville, MD) from healthy subjects and A549 cells were cultured under submerged conditions. SAE cells were used for experiments at passages 3–6 and at a confluency of approximately 70%. Cells were treated with RSV at a multiplicity of infection (MOI) of 0.3 for 24 hours. Immunoreactivity assays were performed on SAE and A549 cells with polyclonal anti-RSV (Abcam; ab20745) antibodies. Cells were also treated with mock control, as described above. Additionally, A549 cells SAE cells were transfected by administering siRNA for MDA5, RIG-I, LGP2, Trif or control siRNA (Qiagen, Gaithersburg, MD).

### Animal models

*Trif* (*Ticam1*) and *Mavs* knockout (KO) mice were purchased from the Jackson Laboratory (Bar Harbor, ME) and bred on to a FVB/NJ background at least 6 generations. All mice were maintained in a specific pathogen-free facility at Mount Sinai Roosevelt Hospital. 8-week-old mice were used at the initiation point for all experiments and each experimental parameter had 10 animals/group. Mice were anesthetized by intraperitoneal (IP) injection of a mixture of ketamine and xylazine. Animals were intranasally administered 1×10^6^ PFU RSV or mock. Animals were euthanized on days 0, 1, 3, 5, 7 and 9 post RSV or mock administering. Bronchoalveolar lavage fluid (BALF) and tissues were collected for cytokine analysis. FVB/NJ mice were IP injected with 100 μg of normal goat IgG (R&D Systems) or a neutralizing goat polyclonal IgG targeting murine LIF (R&D Systems) 2 hours prior to RSV administration. Lung viral titer and RSV N copy number were determined as previously described [69]. BALF cells were analyzed for apoptosis utilizing the LIVE/DEAD cell viability assay from Life Technologies on the Guava easyCyte flow cytometer from Millipore. All animal experiments were performed with approval from Mount Sinai Roosevelt’s Hospital’s Institutional Animal Care and Use Committee approval.

### Cytokine measurements

Mouse IL-4, IL-6, IL-18, IL-22, IL-23, IL-27, CXCL1/KC, CXCL9, CXCL10/IP-10, CXCL11, MIF, TGF-β, CCL2, LIF, CXCL12, CCL27, SCGF-β, SCF, MCP-1/CCL2, RANTES/CCL5 and MIP-1α/CCL3 gene expression was performed by quantitative PCR (qPCR) using Taqman probes (Life technologies/Applied Biosystems, Carlsbad, CA). 42 human and 26 mouse cytokines were examined in human cell media and mouse BALF, respectively, using beads assays (Bio-Rad Magnetic Cytokine Bead Panels, groups 1 and 2) with the BioRad Bio-Plex 200 system (BioRad, Hercules, CA). ELISAs were utilized to determine mouse CXCL12 (R&D systems), CCL27, SCGF-β and SCF (Abcam) BALF levels.

### Intracellular signaling

Cells were lysed in radio-immunoprecipitation assay (RIPA) buffer, centrifuged at 13,000 *× g* for 10 minutes and supernatants collected. Immunoblots were conducted to determine levels of p-IRF3(ser396), IRF3, p-TBK1/NAK(Ser172), TBK1/NAK, RIG-I, MDA5, LGP2, TLR2, TLR3, TLR7, TLR8, TLR9, Trif, MyD88, IRAK1, NOD2 and actin (all antibodies from Cell Signaling Technologies). TLRs, NOD1, NOD2, NXLR1, RIG-I, MDA5, Trif and actin expressions were determined by qPCR using Taqman probes (Life technologies/Applied Biosystems).

### Airway responses to methacholine challenge

Airway responses to methacholine (Sigma Chemical, St. Louis, MO) were assessed with the Scireq Flexivent system (Scireq, Montreal, QC, Canada) 1-week post RSV challenge and IP injection of control IgG or anti-LIF IgG. Animals were anesthetized with ketamine/xylazine (10 mg/kg) and paralysis was induced with 1 mg/kg pancuronium bromide IP (Sigma). The linear single-compartment model was used to assess total respiratory system resistance (Rn). Methacholine dose responses were determined.

### Statistical analyses

For statistical analysis, data from 10 animals or multiple separate cell experiments were pooled. Data are expressed as means ± S.E.M. Differences between groups of mice over time were compared by two-way analysis of variance (ANOVA). Individual differences between groups were tested by multiple comparison and analysis using the Bonferroni post-test. Pairs of groups were compared by Student’s t test (two tailed). p values for significance were set at 0.05. All analysis was performed using GraphPad Prism Software (Version 5 for Mac OS X).

## Additional file

Additional file 1: Figure S1. Profiling A549 cell cytokine response utilizing protein arrays. A549 cells were infected at an MOI of 0.3 for 24 hours. Cytokine arrays were performed with 24-hour media from mock and RSV treated cells. Densitometry were performed. Each pair of horizontal spots represents one cytokine. Graphs are represented as mean pixel intensity ± S.E.M, where n=2 spots/group and each assay were performed on 3 samples/group collects on separate days. **Figure S2.** Quantification of pathogen recognition receptors and kinase immunoblots of RSV treated A549 and SAE cells. (A) SAE and (B) A549 cells were treated with mock or RSV (MOI of 0.3) for 24 hours. Immunoblots (from Figure 3B) were subjected to densitometry analysis. Graphs are represented as mean pixel intensity (ratios of total protein to actin or phosphorylated protein to total protein)± S.E.M, where n=3 replicates/group and each assay were performed on samples obtained on multiple days (3) and from multiple immunoblots. **Figure S3.** Confirmation that expression of Trif and RLRs was silenced in A549 cells. A549 cells were transfected with siRNA targeting RIG-I (DDX58), LGP2 (DHX58), MDA5 (IFIH1), Trif or a negative sequence control. Immunoblots were performed to determine RIG-I, LGP2, MDA5 and Trif protein levels compared to actin.

## Abbreviations

RSV: Respiratory syncytial virus
BALF: Bronchoalveolar lavage fluid
SAE: Small airway epithelial
RIG-I: Retinoic acid–inducible gene-1
MDA5: Melanoma differentiation–associated protein-5
MAVS: Mitochondrial antiviral-signaling protein
RLRs: RIG-I-like receptors
NOD: Nucleotide-binding oligomerization domain-containing protein
LGP2: Laboratory of Genetics and Physiology 2
LIF: Leukemia inhibitory factor
MIF: Migration inhibitory factor
SCF: Stem cell factor (SCF)
SCGF-β: Stem cell growth factor beta
