# Supplementary material for: Leukemia inhibitory factor protects the lung during respiratory syncytial viral infection
Source: BMC Immunol. 2014 Oct 3;15:41. doi: 10.1186/s12865-014-0041-4 (PMC4189665; doi:10.1186/s12865-014-0041-4)

Additional Files

Methods

Human cytokine protein array. Secretion of cytokines after 24 hour RSV infection was assessed by human cytokine protein array (R&D Systems, Minneapolis, MN), which detects the relative levels of 36 different cytokines, chemokines, and acute phase proteins in a single sample as per the manufacturer's instructions.

Supplemental Figure S1. Profiling A549 cell cytokine response utilizing protein arrays. A549 cells were infected at an MOI of 0.3 for 24 hours. Cytokine arrays were performed with 24-hour media from mock and RSV treated cells. Densitometry were performed. Each pair of horizontal spots represents one cytokine. Graphs are represented as mean pixel intensity ± S.E.M, where n=2 spots/group and each assay were performed on 3 samples/group collects on separate days.

Supplemental Figure S2. Quantification of pathogen recognition receptors and kinase immunoblots of RSV treated A549 and SAE cells. (A) SAE and (B) A549 cells were treated with mock or RSV (MOI of 0.3) for 24 hours. Immunoblots (from Figure 3B) were subjected to densitometry analysis. Graphs are represented as mean pixel intensity (ratios of total protein to actin or phosphorylated protein to total protein)± S.E.M, where n=3 replicates/group and each assay were performed on samples obtained on multiple days (3) and from multiple immunoblots.

Supplemental Figure S3. Confirmation that expression of Trif and RLRs was silenced in A549 cells. A549 cells were transfected with siRNA targeting RIG-I (DDX58), LGP2 (DHX58), MDA5 (IFIH1), Trif or a negative sequence control. Immunoblots were performed to determine RIG-I, LGP2, MDA5 and Trif protein levels compared to actin.

Supplemental Figure S1

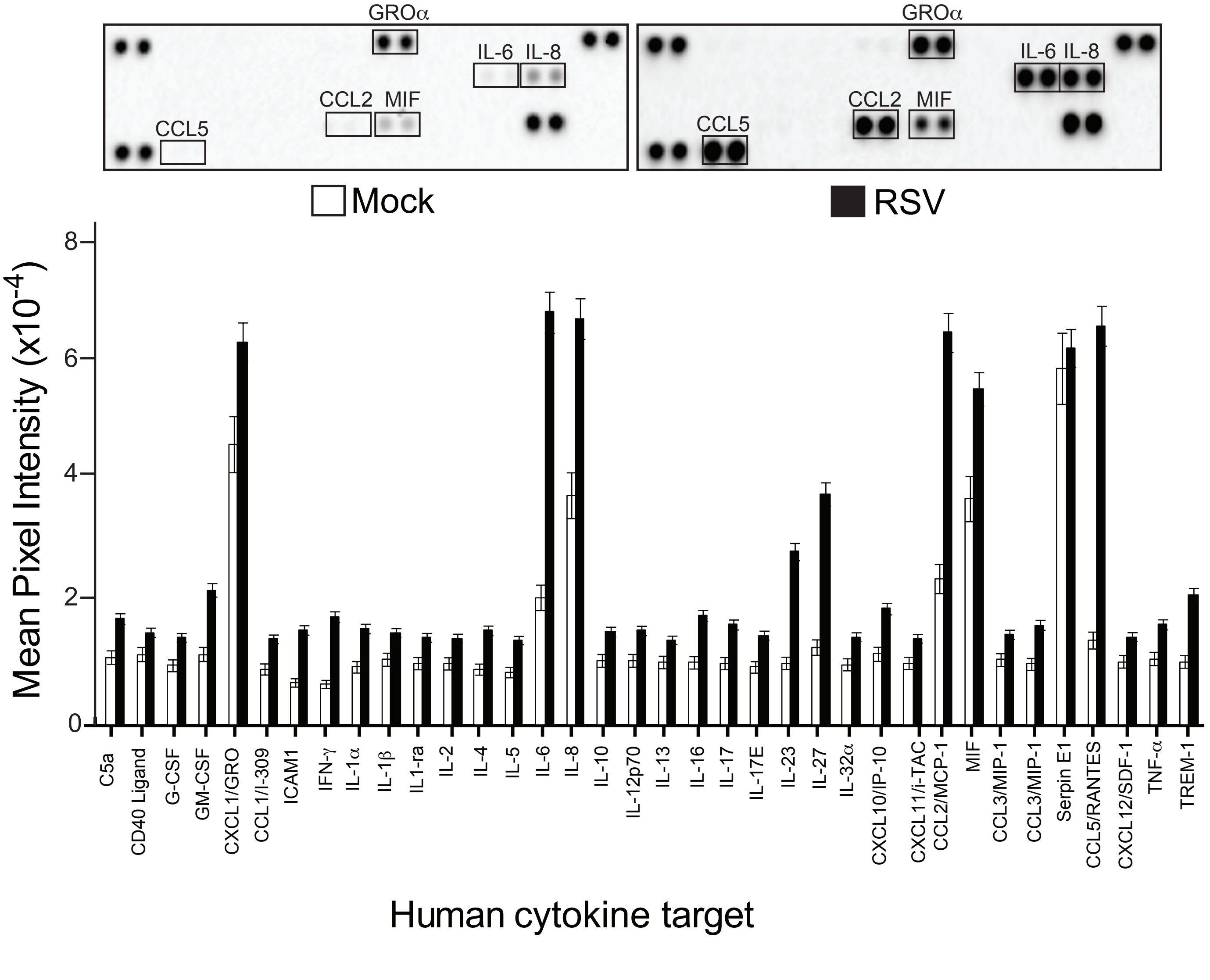

Supplemental Figure S2

A

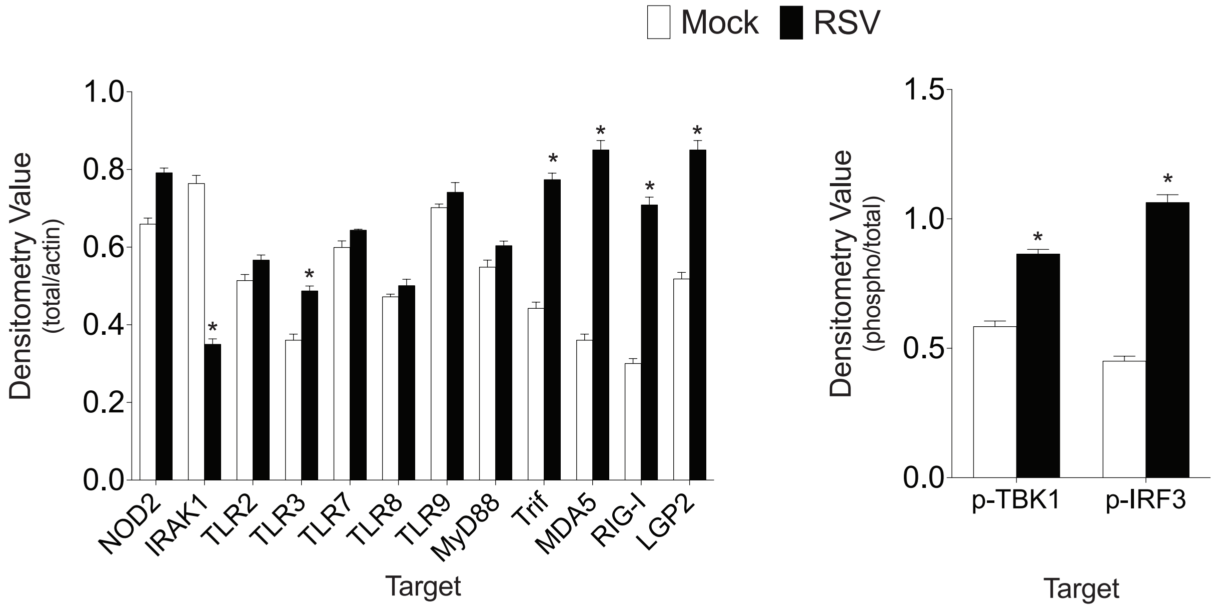

B

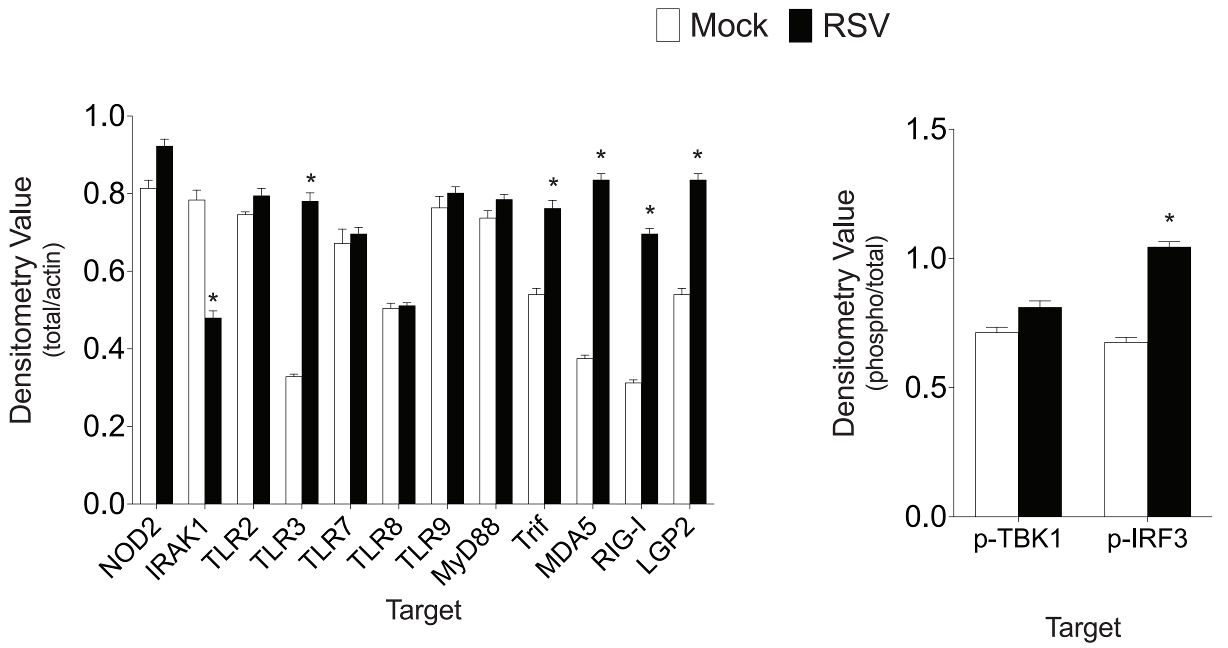

Supplemental Figure S3

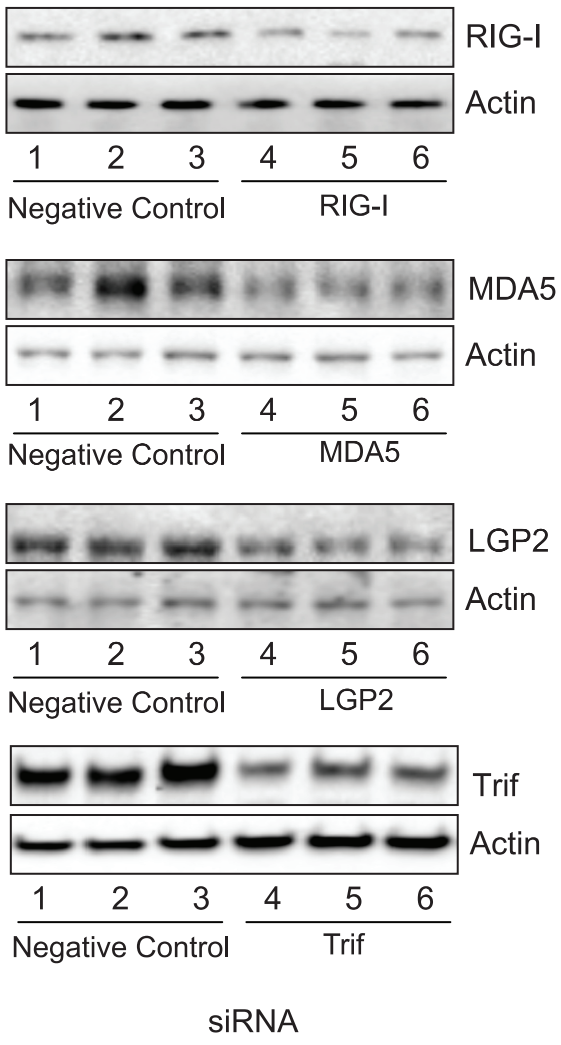

Supplement: Additional file 1: Figure S1. — Profiling A549 cell cytokine response utilizing protein arrays. A549 cells were infected at an MOI of 0.3 for 24 hours. Cytokine arrays were performed with 24-hour media from mock and RSV treated cells. Densitometry were performed. Each pair of horizontal spots represents one cytokine. Graphs are represented as mean pixel intensity ± S.E.M, where n=2 spots/group and each assay were performed on 3 samples/group collects on separate days. Figure S2. Quantification of pathogen recognition receptors and kinase immunoblots of RSV treated A549 and SAE cells. (A) SAE and (B) A549 cells were treated with mock or RSV (MOI of 0.3) for 24 hours. Immunoblots (from Figure 3B) were subjected to densitometry analysis. Graphs are represented as mean pixel intensity (ratios of total protein to actin or phosphorylated protein to total protein)± S.E.M, where n=3 replicates/group and each assay were performed on samples obtained on multiple days (3) and from multiple immunoblots. Figure S3. Confirmation that expression of Trif and RLRs was silenced in A549 cells. A549 cells were transfected with siRNA targeting RIG-I (DDX58), LGP2 (DHX58), MDA5 (IFIH1), Trif or a negative sequence control. Immunoblots were performed to determine RIG-I, LGP2, MDA5 and Trif protein levels compared to actin. [file 12865_2014_41_MOESM1_ESM.pdf]
